# Radiological features of COVID‐19‐associated mucormycosis: A report of 36 cases along with a literature review

**DOI:** 10.1002/ccr3.8154

**Published:** 2023-11-20

**Authors:** Alireza Omranzadeh, Mohamadreza Afzalzadeh, Alireza Ghodsi, Hashem Neshati, Masoud Mahdavi Rashed

**Affiliations:** ^1^ Mashhad University of Medical Sciences Mashhad Iran; ^2^ ENT Department Mashhad University of Medical Sciences Mashhad Iran; ^3^ Radiology Department Mashhad University of Medical Sciences Mashhad Iran

**Keywords:** computed tomography, COVID‐19, diagnostic imaging, mucormycosis

## Abstract

The pandemic of COVID‐19 along with high use of corticosteroids resulted in the emergence of invasive fungal infection. Here, we reported the radiologic feature of mucormycosis in COVID‐19‐infected cases and reviewed with available literature.

## Graphical abstract

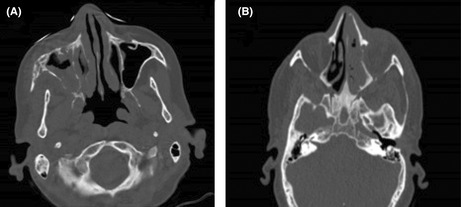

## 1 INTRODUCTION

The global pandemic of coronavirus disease 2019 (COVID‐19) has caused a significant public health concern worldwide,^1^ affecting all age groups.^2^, ^3^ Whereas most of the COVID‐19 patients will have a mild to moderate respiratory disease that will improve without using special medications, the severe form of COVID‐19 is more likely to affect the elderly and those with underlying medical conditions.^4^ In these patients, the infection causes rapid respiratory deterioration, which could also lead to acute respiratory distress syndrome (ARDS).^5^

The fungal and bacterial co‐infections have been identified in patients with influenza, Middle East respiratory syndrome coronavirus, and severe acute respiratory syndrome; however, evidence of co‐infections, specifically fungal infections, among severely ill COVID‐19 patients is scarce.^6^ COVID‐19 patients in severe and critical conditions, who require mechanical ventilation and long stay in the intensive care unit, suffering from ARDS, and who are treated with high doses of corticosteroids, broad‐spectrum antibiotics, interleukin antagonists, and immunomodulators, are at an increased risk of developing fungal infections, including candidemia, pneumocystitis jiroveci pneumonia, aspergillosis, mucosal candidiasis, and mucormycosis.^7^, ^8^, ^9^, ^10^

Mucormycosis is an opportunistic infection that causes infarction and necrosis of end‐organ host tissues, including the skin, orbits, paranasal sinuses, lungs, kidneys, gastrointestinal system, and central nervous system.^11^ A surge in the incidence of invasive rhino‐orbito‐cerebral mucormycosis has been reported worldwide in patients suffering or recovering from COVID‐19 infection,^12^ which can be fatal if not diagnosed early, and involvement of important structures must be carefully investigated employing magnetic resonance imaging (MRI) and computed tomography (CT).^13^, ^14^

There is a paucity of data on the imaging findings of COVID‐19 associated mucormycosis (CAM). On the other hand, effective treatment necessitates prompt diagnosis in order to actively evaluate the areas of involvement and delineate their extensions, which can substantially assist surgical treatment. The present study reports a series of 36 patients with CAM, with the objective of highlighting radiological findings. Furthermore, a review of literature regarding the radiological features of CAM was conducted.

## 2 CASE PRESENTATION

A total of 36 cases were included in our study, including 22 males (61.1%) and 14 females (38.9%). Patients' diagnoses were confirmed through nasal endoscopy and CT scans of the paranasal sinuses. The presence of Mucoralean fungi was confirmed through a complete oral and maxillofacial clinical examination, followed by microbiological and histological evaluations, using biopsy specimens. Histopathology was performed on nasal turbinate/palatal mucosal biopsies using hematoxylin and eosin, periodic acid Schiff, or Gomori methenamine silver stain. Direct microscopy was performed using potassium hydroxide mount. The samples were inoculated on Sabouraud dextrose agar and incubated at 25 and 37°C. Positive cultures were identified by macroscopic and microscopic characteristics and approved by the presence of aseptate hyphae.

The mean age of the patients was 55.56 ± 12.79 years and ranged from 26 to 80 years old. Seven patients (19.4%) had no comorbidities, 19 (52.8%) had diabetes alone, 6 had both diabetes and hypertension, 2 (5.6%) were AML cases, 1 case (2.8%) of concomitant diabetes and immune ITP, and 1 case (2.8%) of concomitant cerebrovascular accident and pulmonary thromboembolism. Moreover, 28 cases (77.8%) received corticosteroid therapy.

In terms of lung involvement, 10 cases (27.8%) had <10% involvement and 14 cases (38.9%) had 50% or greater involvement. Most of the cases (25 patients; 69.4%) had pan‐sinusitis, followed by 5 cases (13.9%) of maxillary‐ethmoid‐sphenoid involvement, 5 cases (13.9%) of maxillary‐ethmoid involvement, and 1 case (2.8%) of ethmoid‐sphenoid affection. Twelve cases (33.3%) had bilateral, 8 (22.2%) had left, and 5 (13.8%) had right pan‐sinusitis. Also, there was one bilateral case (2.7%), one left case (2.7%), and three (8.3%) right cases of maxillary‐ethmoid‐sphenoid involvement. Furthermore, among maxillary‐ethmoid involvement cases, 2 (5.5%) were on the left and 3 (8.3%) were on the right side. The only case of ethmoid‐sphenoid involvement was on the right side. Moreover, 11 cases (30.6%) had middle concha affection and 25 cases (69.4%) had middle‐inferior concha affection.

**FIGURE 1:**
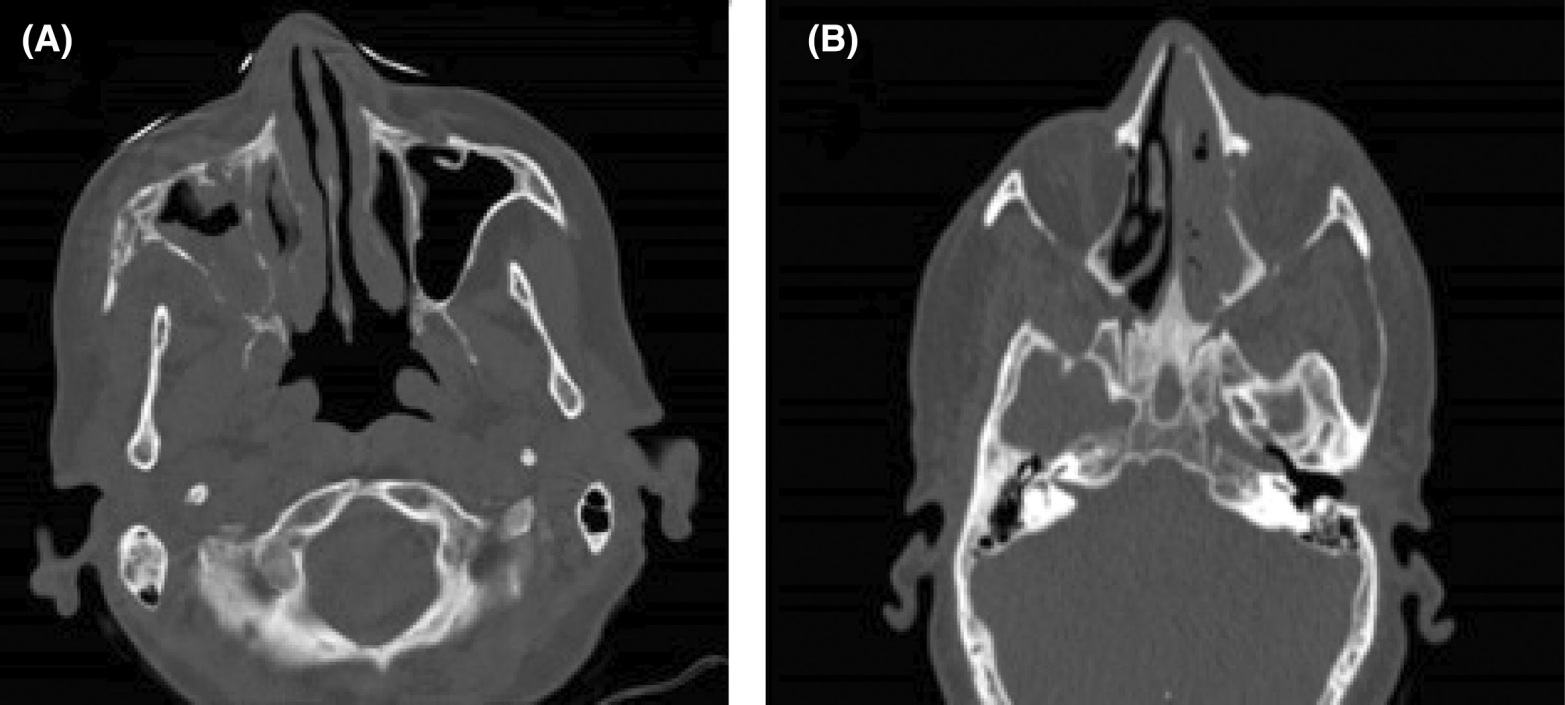
(A) Mucormycosis involvement of the right maxillary sinus and middle turbinate along with erosions of sinus walls in CT scan. (B) Opacity in left ethmoidal sinus and nasal cavity with lamina papyracea destruction and orbital involvement in CT scan.

According to CT scan findings, retro‐antral fat stranding was present in 30 cases (83.3%), 34 cases (94.4%) had sinus bone erosion, and 6 cases (16.7%) had bone destruction. Figure 1A,B show bone erosion and destruction, respectively. Hyperdense sinus was evident in 7 cases (19.4%). Only 5 cases (13.8%) had none of the kinds of orbital cellulitis, 26 patients (72.2%) showed both preseptal and postseptal cellulitis, and 5 patients (13.8%) only showed preseptal cellulitis. Proptosis was also present in 25 out of 26 (96.1%) postseptal cellulitis patients.

**FIGURE 2:**
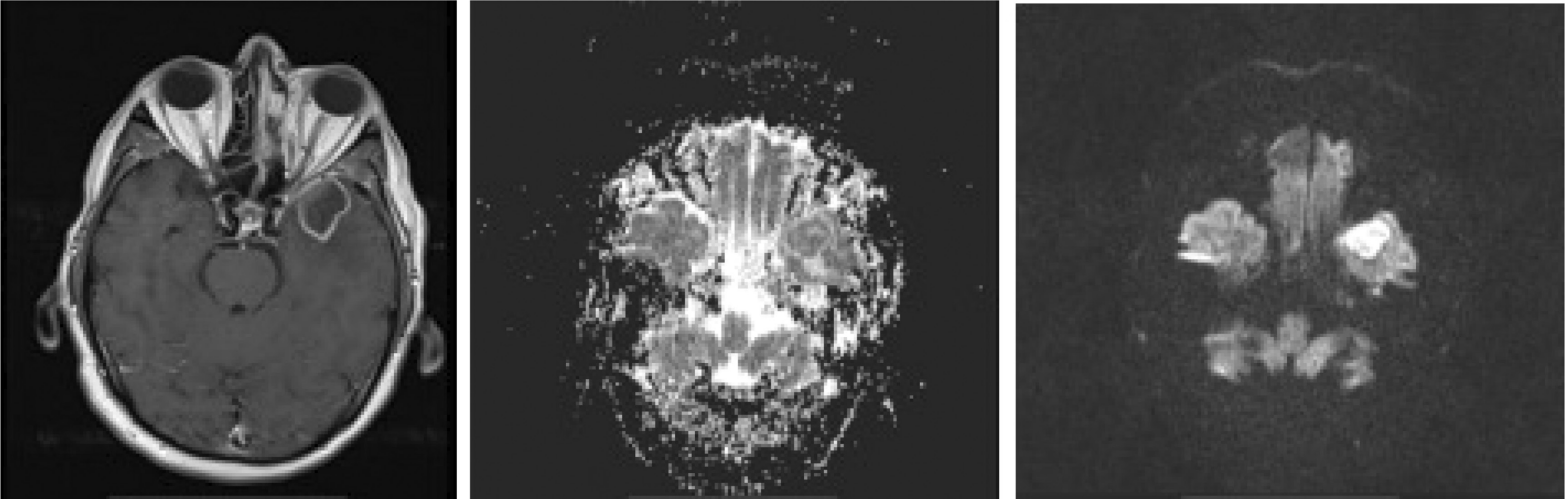
Rim enhancing abscess in left temporal lobe with restriction in DWI sequence.

**FIGURE 3:**
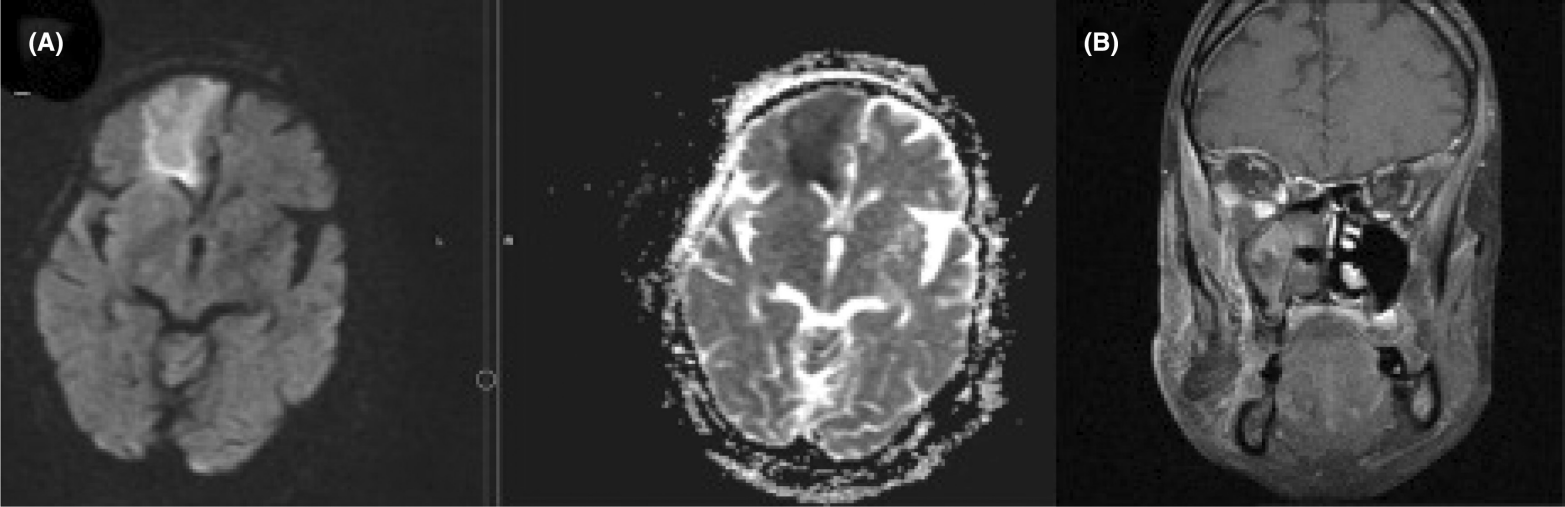
(A) Infarction in the right frontal lobe due to Willis circle infiltration with mucormycosis. (B) T1 coronal sequence with contrast study, showing black turbinate sign in the right middle and inferior conchae.

MRI with contrast injection was conducted to assess brain involvement and collection formation (Figure 3A). Six cases (16.6%) had collection, including 2 cases (5.6%) of subperiosteal abscess medial to ethmoid, 1 case (2.8%) of subperiosteal abscess with interaorbital extension, 1 case (2.8%) of bilateral subperiosteal abscess medial to ethmoid, 1 case (2.8%) of preseptal collection, and 1 case of left masticator space abscess along with temporal abscess. The MRI study of the only case of temporal abscess is demonstrated in Figure 2. Brain infarction was evident in 4 cases (11.1%). Moreover, 5 cases (13.9%) had intracranial vessel involvement, 5 cases (13.9%) had cavernous sinuous involvement, and 2 cases (5.6%) had internal carotid involvement. In cases of dura involvement, 6 cases (16.7%) showed a pachymeningeal pattern and 1 case (2.8%) had a leptomeningeal pattern. Also, the black turbinated sign, which is demonstrated in an MRI section with contrast study in Figure 3B, was present in 30 cases (83.3%). Furthermore, 30 cases (83.3%) had black turbinate signs.

The antifungal agents, which were used to treat mucormycosis, were dependent on the severity of the infection and the patient's overall health. But we commonly used liposomal amphotericin with a dose of 5 mg/kg per day. In patients with orbital or cerebral involvement, we used 10 mg/kg per day liposomal amphotericin. Serial endoscopic sinus cavity debridement was done for all patients. However, the extent of surgery required may depend on the severity of the infection and the extent of tissue damage. Orbital exenteration was performed for two patients. All patients had negative blood culture. The pulmonary thromboembolism in one patient conflicted was treated medically. No gastrointestinal lesion was detected in patients.

**FIGURE 4:**
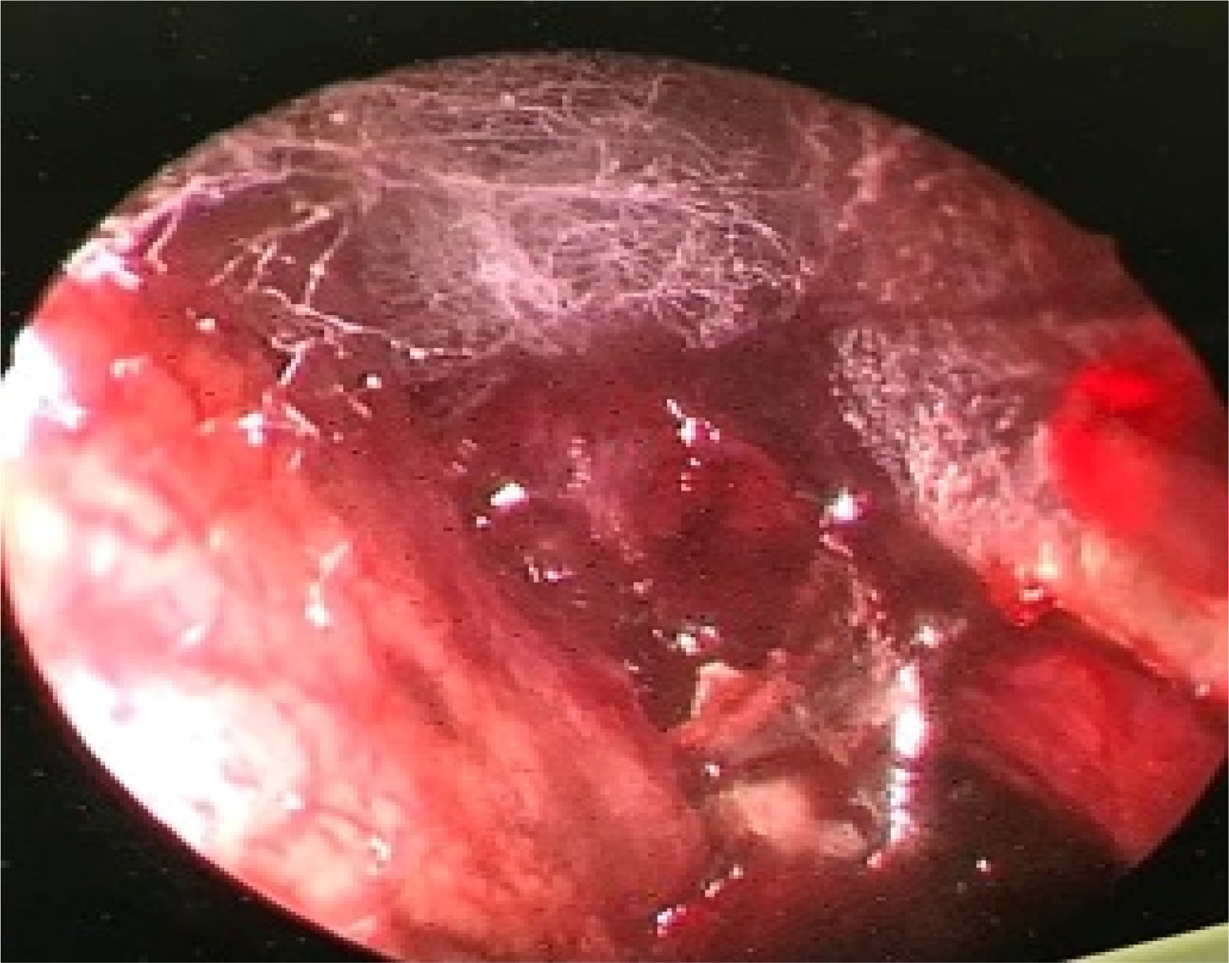
Mucor hyphae resected using minimally invasive endoscopic surgery.

All the patients received systemic antifungal medication along with surgical removal of the hyphae. At the final follow‐up, unfortunately, 14 cases succumbed to the disease, and thus the mortality rate was 38.9%. Figure 4 reveals the *Mucor* hyphae during endoscopic surgery. Also, the details of all cases are demonstrated in Table 1.

**TABLE 1:** Baselines, CT, and MRI findings of the each of the cases.

Baselines					Severity of Lung involvement (%)	CT findings										MRI findings								Outcome
Patient	Gender	Age	Comorbidity	Corticosteroid usage for COVID‐19 treatment		Side	Concha erosion involvement	Retro‐antral fat stranding	Sinus involvement	Hyper‐ dense sinus content	Sinus Bone Erosion	Bone Destruction	Orbital cellulitis (postseptal cellulitis)	Periorbital cellulitis (preseptal cellulitis)	Proptosis	Brain Infarction	Intracranial vessel involvement	Leptomeningeal enhancement	Pachymeningeal enhancement	Cavernous sinus involvement	Carotid involvement	Black Turbinate Sign	Collection (intra cranial or other spaces)	
1	F	50	DM	N	30	L	Middle‐Inferior	P	Pan‐sinusitis	N	P	N	P	P	P	P	N	N	N	N	N	P	N	Alive
2	M	41	DM	P	10	L	Middle‐Inferior	P	Pan‐sinusitis	P	P	N	P	P	P	N	N	N	N	N	N	P	**P** ^a^	Alive
3	F	55	DM‐ITP	P	60	R	Middle‐Inferior	P	Pan‐sinusitis	P	P	N	P	P	P	N	N	N	N	N	N	P	N	Death
4	M	69	DM	P	40	L	Middle‐Inferior	P	Maxillary‐ethmoid‐sphenoid	P	P	P	P	P	P	N	P	N	N	P	N	P	N	Alive
5	F	74	DM	P	20	L	Middle‐Inferior	P	Pan‐sinusitis	N	P	N	N	P	N	N	N	N	N	N	N	P	N	Alive
6	M	80	DM‐HTN	P	50	L	Middle‐Inferior	P	Pan‐sinusitis	P	P	N	P	P	P	N	N	N	P	N	N	P	N	Death
7	M	77	DM‐HTN	P	50	L	Middle‐Inferior	P	Pan‐sinusitis	P	P	N	P	P	P	N	N	N	N	N	N	P	**P** ^b^	Alive
8	F	68	DM	N	<10	B	Middle	N	Pan‐sinusitis	N	P	N	N	N	N	N	N	N	N	N	N	P	N	Alive
9	M	64	DM	P	30	L	Middle‐Inferior	P	Pan‐sinusitis	N	P	N	P	P	P	N	N	N	P	N	N	P	N	Alive
10	F	56	None	N	60	R	Middle‐Inferior	P	Maxillary‐ethmoid‐sphenoid	N	P	N	P	P	P	N	P	N	N	P	N	P	**P** ^b^	Death
11	M	35	DM	P	<10	R	Middle	P	Maxillary‐ethmoid	N	P	N	P	P	P	N	N	N	N	N	N	P	N	Alive
12	M	72	DM‐HTN	P	25	B	Middle	P	Pan‐sinusitis	N	P	N	P	P	P	P	N	N	N	N	N	P	N	Death
13	M	51	DM‐HTN	P	20	B	Middle	N	Pan‐sinusitis	N	N	N	N	N	N	P	N	N	P	N	N	P	N	Death
14	M	58	CVA‐PTE	P	80	R	Middle	P	Maxillary‐ethmoid‐sphenoid	N	P	N	P	P	P	P	P	P	P	P	P	P	N	Death
15	M	43	None	P	30	R	Middle‐Inferior	P	Pan‐sinusitis	P	P	N	N	P	N	N	N	N	N	N	N	N	N	Alive
16	M	57	AML	P	90	L	Middle‐Inferior	P	Pan‐sinusitis	N	P	P	P	P	P	N	N	N	N	N	N	P	N	Death
17	M	26	None	N	10	R	Middle‐Inferior	N	Maxillary‐ethmoid	N	P	N	N	N	N	N	N	N	N	N	N	N	N	Alive
18	F	60	DM‐HTN	P	<10	B	Middle‐Inferior	P	Pan‐sinusitis	N	P	P	P	P	P	N	N	N	N	N	N	P	**P** ^c^	Alive
19	F	46	DM	P	<10	B	Middle	P	Pan‐sinusitis	N	P	N	N	P	N	N	N	N	N	N	N	N	N	Alive
20	M	46	DM	P	50	B	Middle‐Inferior	N	Pan‐sinusitis	N	P	N	P	P	P	N	N	N	N	N	N	P	N	Alive
21	M	54	AML	P	20	B	Middle‐Inferior	N	Pan‐sinusitis	N	P	N	N	P	N	N	N	N	N	N	N	N	N	Alive
22	M	28	None	N	<10	R	Middle‐Inferior	P	Pan‐sinusitis	N	P	P	N	N	N	N	N	N	N	N	N	P	N	Alive
23	M	65	None	N	<10	R	Middle	N	Ethmoid‐sphenoid	N	N	N	N	N	N	N	N	N	N	N	N	N	N	Alive
24	F	52	DM	P	60	B	Middle‐Inferior	P	Pan‐sinusitis	N	P	N	P	P	P	N	N	N	N	N	N	P	**P** ^d^	Death
25	F	64	None	P	80	L	Middle‐Inferior	P	Pan‐sinusitis	N	P	N	P	P	P	N	N	N	N	N	N	P	N	Death
26	M	53	DM	P	80	B	Middle	P	Pan‐sinusitis	N	P	P	P	P	P	N	N	N	N	N	N	P	N	Death
27	F	60	DM	P	60	B	Middle‐Inferior	P	Pan‐sinusitis	N	P	N	P	P	P	N	N	N	N	N	N	P	N	Death
28	F	51	DM	P	60	R	Middle‐Inferior	P	Pan‐sinusitis	N	P	N	P	P	P	N	N	N	N	N	N	P	N	Death
29	M	62	DM	P	10	R	Middle	P	Pan‐sinusitis	N	P	N	P	P	P	N	N	N	N	N	N	P	N	Death
30	M	70	None	N	<10	B	Middle‐Inferior	P	Pan‐sinusitis	N	P	N	N	P	N	N	N	N	N	N	N	N	N	Alive
31	F	63	DM	P	<10	R	Middle	P	Maxillary‐ethmoid	N	P	P	P	P	P	N	N	N	N	N	N	P	N	Alive
32	F	63	DM‐HTN	N	<10	R	Middle	P	Maxillary‐ethmoid‐sphenoid	P	P	N	P	P	P	N	N	N	N	N	N	P	N	Alive
33	M	44	DM	P	10	L	Middle‐Inferior	P	Maxillary‐ethmoid	N	P	N	P	P	P	N	P	N	P	P	P	P	N	Alive
34	M	42	DM	P	70	B	Middle‐Inferior	P	Maxillary‐ethmoid‐sphenoid	N	P	N	P	P	P	N	N	N	N	N	N	P	N	Death
35	F	53	DM	P	<10	L	Middle‐Inferior	P	Maxillary‐ethmoid	N	P	N	P	P	N	N	P	N	P	P	N	P	N	Alive
36	M	48	DM	P	50	B	Middle‐Inferior	P	Pan‐sinusitis	N	P	N	P	P	P	N	N	N	N	N	N	P	**P** ^e^	Alive

Abbreviations: AML, acute myeloid leukemia; B, bilateral; CVA, cerebrovascular accident; DM, diabetes; F, female; HTN, hypertension; ITP, immune thrombocytopenia; L, left; M, male; N, negative; P, positive; PTE, pulmonary thromboembolism; R, right.

^a^ Preseptal collection.

^b^ Subperiosteal abscess medial to ethmoid.

^c^ Subperiosteal abscess with interaorbital extension.

^d^ Bilateral subperiosteal abscess medial to ethmoid.

^e^ Left masticator space abscess along with temporal abscess.

## 3 DISCUSSION

The rise in mucormycosis superinfection during the COVID‐19 pandemic, yielded in the terms of CAM. The increase in the number of infections due to this fungus was mainly during the rise of delta variants of COVID‐19. In fact, the mutation made the virus more infective, virulent, and even invaded the body's immune system. Along with the immunodeficiency caused by this virus, the number of severe cases is far higher with the delta variant, and thus the use of systemic steroids becomes higher in these cases. All these, along with the fact that most of the severe cases of COVID‐19 have many underlying diseases like diabetes, vindicate the epidemic of CAM during the delta mutation pandemic.^15^ In fact, immunosuppression is the key element that helps mucormycosis to outface.^16^ In line with this proposal, 77.8% of our cases received systemic corticosteroids and 72.2% were diabetic. A literature review of 10 studies showed that the use of glucocorticoids and the prevalence of diabetes in CAM cases ranged from 40%^17^ to 100%^18^, ^19^ and 52.2%^20^ to 100%.^17^ respectively. Table 2 demonstrates a thorough literature review of these 10 studies.

**TABLE 2:** The review of demographics, risk factors, mortality, and imaging findings of COVID‐19 associated mucormycosis.

Study	Number of cases (*N*)	Age (mean; years)	Gender (male; *N* %)	Diabetes (*N*; %)	Corticosteroid (*N*; %)	Side			Sinus involvement							Orbital involvement		MRI						Mortality rate (*N*; %)
						Right	Left	Both	Maxillary	Ethmoid	Sphenoid	Frontal	Bone erosion	Hyper‐density	Retro‐antral fat stranding	Totally	Proptosis	Infarction	Pachymeningeal involvement	Cavernous sinuous involvement	Carotid involvement	Collection (intra cranial or other spaces)	Black turbinate	
Joshi et al.^18^	25	55.2	16 (64.0)	22 (61.1)	25 (100)	—	—	—	25 (100)	19 (76.0)	10 (40.0)	8 (32.0)	20 (80.0)	6 (24.0)	18 (72.0)	23 (92.0)	—	5 (20.0)	2 (8.0)	9 (36.0)	—	—	8 (32.0)	14 (56.0)
Yadav et al.^21^	50	49.5	31 (62.0)	43 (86.0)	22 (44.0)	—	—	—	46 (92.0)	46 (92.0)	41 (82.0)	28 (56.0)	42 (84.0)	32 (64.0)	37 (74.0)	38 (76.0)	27 (54.0)	8 (16.0)	19 (38.0)	16 (32.0)	8 (16.0)	15 (30.0)	41 (82.0)	—
Desai et al.^13^	50	23–73^a^	29 (58.0)	42 (84.0)	42 (84.0)	22 (44.0)	20 (40.0)	31 (62.0)	26 (52.0)	19 (38.0)	6 (12.0)	0 (0.0)	42 (84.0)	—	—	35 (70.0)	—	—	21 (42.0)	18 (36.0)	—	—	—	15 (30.0)
Selarka et al.^19^	47	55	35 (74.5)	36 (76.6)	47 (100.0)	—	—	—	47 (100.0)	35 (74.5)	36 (76.6)	62 (68.1)	—	—	—	19 (40.4)	—	5 (10.6)	—	2 (4.2)	1 (2.1)	1 (2.1)	—	11 (23.4)
Ravani et al.^29^	31	56.3	20 (64.5)	30 (96.7)	19 (61.2)	—	—	—	25 (80.6)	26 (83.8)	26 (83.8)	24 (77.4)	—	—	—	19 (61.2)	—	—	—	1 (3.2)	2 (6.4)	—	—	3 (9.6)
Kumari et al.^31^	20	53.9	11 (55.0)	16 (80.0)	16 (80.0)	—	—	—	16 (80.0)	18 (90.0)	8 (40.0)	7 (35.0)	—	—	—	11 (55.0)	—	—	—	—	—	3 (15.0)	—	6 (30.0)
Sen et al.^23^	2826	51.9	1993 (71.0)	2194 (78.0)	2073 (87.0)	1585 (60.1)		1050 (39.9)	2180 (90.0)	1925 (79.0)	1498 (61.5)	1426 (58.5)	—	—	—	1713 (64.2)	—	—	—	285 (10.6)	95 (3.5)	81 (3.0)	—	395 (14.0)
Singhal et al.^20^	25	17–78^a^	15 (60.0)	13 (52.0)	25 (100.0)	—	—	—	24 (96.0)	20 (80.0)	18 (72.0)	12 (48.0)	2 (8.0)	0 (0.0)	16 (89.0)	15 (60.0)	13 (52.0)	1 (4.0)	3 (12.0)	2 (8.0)	2 (8.0)	3 (12.0)	5 (20.0)	6 (24.0)
Patel et al.^30^	96	49.39^b^	73 (76.0)	69 (71.9)	79 (82.3)	29 (30.3)		67 (69.7)	92 (95.8)	88 (91.6)	75 (78.2)	61 (63.6)	72 (75.0)	32 (30.3)	49 (51.0)	57 (59.4)	—	6 (6.2)	8 (8.3)	7 (7.3)	5 (5.2)	5 (5.2)	—	—
Nehara et al.^17^	5	62.2	4 (80.0)	5 (100.0)	2 (40.0)	3 (60.0)	1 (20.0)	1 (20.0)	5 (100.0)	5 (100.0)	5 (100.0)	4 (80.0)	1 (20.0)	—	—	4 (80.0)	—	1 (20.0)	—	3 (60.0)	—	—	—	2 (40.0)
Our study	36	55.5	22 (61.1)	26 (72.2)	28 (77.8)	12 (33.3)	11 (30.6)	13 (36.1)	35 (97.2)	36 (100.0)	31 (86.1)	25 (69.4)	34 (94.4)	7 (19.2)	30 (83.3)	31 (86.1)	25 (69.4)	4 (11.1)	6 (16.7)	5 (13.9)	2 (5.6)	6 (16.7)	30 (83.3)	14 (38.9)

Abbreviations: B, bilateral; R, right; L, left.

^a^ Reported as age range and not mean age.

^b^ Reported as median age and not mean age.

In terms of demographics, the mean age of our cases was 55.5 years old. Similarly, the reported mean age among the reviewed studies was 49.5^21^ to 62.2^17^ years old. In fact, it is reported that most peoples in their 40s or 50s are more prone to CAM.^16^, ^22^ Furthermore, males are more susceptible to CAM than females, as 61.1% of our patients were men, and this ranged from 58%^13^ to 80%^17^ among various reviewed studies. This may be partly due to the higher prevalence and severity of COVID‐19 infection in the male gender and also to the higher outdoor exposure of men to the fungal spores.^23^, ^24^

It is reported that spores are transferred to the nasal area through inhalation, causing rhino‐sinusitis. However, there are reports of other body parts involvement with CAM, including the lower respiratory system and gastrointestinal tract.^25^ In this regard, a typical CAM case is predominantly a diabetic male who received corticosteroid therapy and is aged between 40 and 60 years old. This patient presents with sino‐nasal involvement signs and symptoms including acute sinusitis, headache, nasal and sinus pain, unexpected toothache, blackish discharge, and prolonged fever irresponsive to antibiotics.^16^

When the suspicion of CAM is developed, the next step is imaging and biopsy assessment for the final diagnosis and differentiation from other invasive fungal infections. Although the gold standard for CAM diagnosis is pathology assessment, radiology evaluation, including CT scan and MRI is used for the assessment of the extent of the infection and surgical planning. Moreover, improvement in the imaging markers helps avoid invasive interventions for diagnosis.^25^, ^26^ In this regard, a thorough study on the imaging findings of CAM is really important.

The modality of choice for CAM cases is plain CT scan of the paranasal sinuses along with contrast‐enhanced brain MRI. In fact, a CT scan provides a better view of hard tissue involvement and MRI has a better contrast for soft tissue assessment.^13^ *Mucor* usually enters the upper respiratory system via inhalation and settles in the nasal mucosa, and involving the middle turbinate, which is known to be the most common site of involvement.^27^ In line with this fact, all of our cases had middle concha involvement.

After the long trip of the spores and their settlement in the nasal mucosa, they start their short trip to the adjacent air‐sinuses. Two of the most commonly involved sinuses are the maxillary and ethmoid, followed by the sphenoid sinus. The frontal sinus is the least involved airspace.^28^ Our study showed that the maxillary sinus was involved in 97.2%, the ethmoid in 100%, the sphenoid in 86.1%, and the frontal sinus in 69.4%. The rate of maxillary, ethmoid, sphenoid, and frontal sinus involvement across the studies was 52%–100%,^13^, ^17^, ^18^, ^19^ 38%–100%,^13^, ^17^ 12%–100%,^13^, ^17^ and 0%–80%,^13^, ^17^ respectively.

One of the most common complications of CAM is bony erosion. Although it is hard, a CT scan can assess bony erosions in different affected bones, including the sinus wall.^13^ We found that 94.4% of our cases had sinus wall bone erosion, that ranged between 20% and 84%, among different studies. Sinus wall erosion may be subtle and needs an exact assessment by the radiologist to identify.^13^ Still, further progression of the infection can result in sinus wall destruction,^28^ as 16.7% of our cases had this finding.

Another finding of mucormycosis in a CT scan is sinus hyperdensity; however, absence of this clue does not rule out this fungal infection.^28^ We found that 19.2% of our cases had hyperdense sinus; this was reported to be as high as 64.0% in a study by Yadav et al.^21^; however, hyperdensity was not evident in any of the cases of Singhal et al.^20^ study. Hyperdensity is developed due to the production of metallic waste by the fungus, and therefore, the longer the time of infection, the higher incidence of hyperdense sinus.^13^

Mucormycosis is characterized by invasive, fulminant local and even systemic dissemination. Bone destruction partly helps the spread of the infection; however, this is not the only way that *Mucor* extends to the other body parts. The plant pathogen can travel through vessels and nerves and affect other adjacent part, even the brain.^28^

Due to the presence of the thin lamina papyracea and a complex interwoven venous system, *Mucor* can travel to the orbit and develop preseptal and postseptal cellulitis.^28^ Moreover, ethmoid and/or frontal involvement and the affection of the nasolacrimal duct further help the movement of the pathogen toward the orbit.^27^ The total rate of orbital involvement in our study was 86.1% and ranged between 40.4%^19^ and 92.0%^18^ among different studies. Moreover, 72.2% showed both preseptal and postseptal cellulitis, and 13.9% had only preseptal cellulitis. Furthermore, varying degrees of proptosis are expected in patients with postseptal CAM.^28^ We reported that 69.4% of the cases had proptosis. Similarly, Yadav et al.^21^ and Singhal et al.^20^ reported proptosis relative prevalence of 54% and 52%, respectively.

*Mucor* can ascend to the brain through the cribriform plate, superior orbital fissure, neuroinvasive, and angioinvasive pathways. The parenchyma of the brain, the meningeal layer, and the vascular system may be affected. Microinvolvement of the brain vessels can lead to brain infarction,^28^ which was found in the MRI assessment of 11.1% of our cases and 4%^20^ to 20%^17^, ^18^ of the patients in various studies. Moreover, pachymeningeal involvement was 8%^18^ to 42%^13^ among the reviewed studies and 16.7% in our study. We found no report of the leptomeningeal involvement rate, which was evident in only one case (2.8%) in our study.

The angiotropism of the pathogen presents itself in the central nervous system as cavernous sinus and internal carotid involvement.^26^ In fact, this case‐series reported a rate of 13.9% for cavernous sinus and 5.6% for internal carotid involvement. Studies also reported cavernous sinus involvement of 3.2%^29^ to 60%^17^ and internal carotid involvement of 2.1%^19^ to 16%.^21^

Besides, the angioinvasive pattern presents itself as the so called “black turbinate” sign in the contrast enhanced T 1—weighted MRI sequence, which is indicative of necrotic tissue in this part. This is believed to be one of the early imaging findings of CAM.^27^ A contrast study of our cases showed that 83.3% of the patients had this MRI finding. This was similar to the reported rate by Yadav et al.,^21^ which was 82%; however, unfortunately few studies focused on this important sign.^18^, ^20^

Another important finding that helps early recognition of the disease is retroantral fat stranding or retroantral fat pad.^30^ In fact, the microvascular infiltration of the fungus causes this condition and is reported to be present in 51%^30^ to 89%^20^ of the CAM cases. This rate was 83.3% in our study. Still, little investigation in the case of this finding is available.^18^, ^20^, ^21^, ^30^ Table 2 summarizes all the discussed findings.

Treatment of CAM is mainly based on systemic anti‐fungal therapy along with surgical removal of the *Mucor* hyphae. The important part in the management of these patients is the timely diagnosis,^13^ and in this respect, our study provides valuable clues. Still, the mortality rate of CAM was reported to be 9.6%^29^ to 56%^18^ depending on the follow up time and the expertise of the medical centre. We reported a mortality rate of 38.9% in our multicenter case study.

Our study has some limitations. Making a conclusion based on a retrospective case‐series is not very exact; however, there are limited case‐series like our study, as addressed in the literature review. In this regard, our study provided useful findings along a thorough literature. This can be addressed as a strength. Still, it would be better to assess the relationship of radiologic findings with patients' outcome. Provision of the results of a follow‐up image would be helpful; however, this must be addressed as another restriction of our study.

In conclusion, more focus on the imaging findings of CAM should be imposed and future studies are needed to improve the in‐time diagnosis and better management of CAM cases. In fact, although each of the proposed imaging findings is nonspecific for CAM diagnosis by itself, maybe when different puzzle pieces come together, the final diagnosis can be made as soon as possible.

## AUTHOR CONTRIBUTIONS

**Alireza Omranzadeh:** Data curation; formal analysis; methodology; project administration; writing – original draft. **Mohamadreza Afzalzadeh:** Project administration; resources; writing – review and editing. **Alireza Ghodsi:** Project administration; writing – original draft. **Hashem Neshati:** Project administration. **Masoud Mahdavi Rashed:** Supervision; writing – review and editing.

## 4 FUNDING INFORMATION

None.

## CONSENT STATEMENT

Written informed consent was obtained from the patient or their legal guardians to publish this report in accordance with the journal's patient consent policy.

## Data Availability

The dataset generated and analyzed during the current study are available from the corresponding author upon reasonable request and in accordance with ethical issues.
